# *Janthinobacterium* sp. Strain SLB01 as Pathogenic Bacteria for Sponge *Lubomirskia baikalensis*

**DOI:** 10.3390/pathogens12010008

**Published:** 2022-12-21

**Authors:** Lubov Chernogor, Marina Eliseikina, Ivan Petrushin, Ekaterina Chernogor, Igor Khanaev, Sergei I. Belikov

**Affiliations:** 1 Limnological Institute, Siberian Branch of the Russian Academy of Sciences, 664033 Irkutsk, Russia; 2 A.V. Zhirmunsky National Scientific Center of Marine Biology, Far Eastern Branch, Russian Academy of Sciences, 690041 Vladivostok, Russia; 3 Faculty of Business Communication and Informatics, Irkutsk State University, 664033 Irkutsk, Russia

**Keywords:** sponge *L. baikalensis*, *Janthinobacterium* sp. strain SLB01, violacein, biofilm, pathogen, complete genomes

## Abstract

Sponges (phylum Porifera) are ancient, marine and inland water, filter feeding metazoans. In recent years, diseased sponges have been increasingly occurring in marine and freshwater environments. Endemic freshwater sponges of the Lubomirskiidae family are widely distributed in the coastal zone of Lake Baikal. The strain *Janthinobacterium* sp. SLB01 was isolated previously from the diseased sponge *Lubomirskia baikalensis* (Pallas, 1776), although its pathogenicity is still unknown. The aim of this study was to confirm whether the *Janthinobacterium* sp. strain SLB01 is the pathogen found in Baikal sponge. To address this aim, we infected the cell culture of primmorphs of the sponge *L. baikalensis* with strain SLB01 and subsequently reisolated and sequenced the strain *Janthinobacterium* sp. PLB02. The results showed that the isolated strain has more than 99% homology with strain SLB01. The genomes of both strains contain genes vioABCDE of violacein biosynthesis and floc formation, for strong biofilm, in addition to the type VI secretion system (T6SS) as the main virulence factor. Based on a comparison of complete genomes, we showed the similarity of the studied bacterial strains of *Janthinobacterium* spp. with the described strain of *Janthinobacterium lividum* MTR. This study will help expand our understanding of microbial interactions and determine one of the causes in the development of diseases and death in Baikal sponges.

## 1. Introduction

Sponges (phylum Porifera) are ancient, multicellular metazoans [1]. These organisms are sessile, filter feeding invertebrates living in marine and freshwater ecosystems and contain symbiotic microorganisms such as bacteria, viruses, archaea, cyanobacteria, microalgae, fungi, and protozoa [2,3,4,5,6,7,8,9]. Freshwater sponges (Demospongiae, Spongillida, and Lubomirskiidae) dominate the fauna of the littoral zone of Lake Baikal [10,11]. Endemic Baikal sponges live in symbiosis with various species of eukaryote and prokaryote microorganisms, including chlorophyll-producing algae [12,13,14]. In recent years, several reports were published on diseases and the mortality of endemic sponges in Lake Baikal [15,16,17]. The number of *L. baikalensis* sponges that are most susceptible to disease has decreased significantly [16]. Annually, up to 10–20% of diseased sponges are observed to die during the winter period [16]. Currently, diseased and dying sponges have been observed in many Lake Baikal areas [15,16]. However, the etiology and ecology of the disease and death of sponges remains unknown.

Earlier, an analysis of 16S rRNA gene amplicons revealed a significant increase in the number of opportunistic microorganisms, including *Betaproteobacteria* of the *Oxalobacteraceae* family, in diseased freshwater sponges and in the cell culture of primmorphs [17,18]. Moreover, we isolated, sequenced, and analyzed the genome of the strain *Janthinobacterium* sp. SLB01 from diseased sponges collected from Lake Baikal [19,20,21]. It is known that the bacteria of the *Janthinobacterium* sp. strain SLB01 can grow, form biofilms, and produce the violet pigment violacein and has proteolytic, lipolytic, and saccharolytic activities. In addition, bacteria contains genes encoding violacein, alpha-amylases, phospho-lipases, a type VI secretion system, and proteins for the synthesis and secretion of polysaccharides [19,21]. The strain *Janthinobacterium* sp. SLB01 is a psychrotolerant, violacein–producing, rod-shaped, Gram-negative, and aerobic bacterium that contains violacein pigment and can form a floc [19]. We identified five genes encoding the vioA, vioB, vioC, vioD and vioE of violacein biosynthesis, which may be a pathogenic factor in the bacteria, similar to the identified genes in the published *J. lividum* MTR strain [21,22]. It is known that the bacteria produce the pigment violacein, which is a compound with antibiotic and antiviral properties [23,24]. In addition, it is known that the genus *Janthinobacterium* has a wide occurrence, including in soil, aquatic sites, marine habitats, and high-altitude environments, with a unique ability to survive and colonize new environments [25,26,27] In the strain *Janthinobacterium* sp. SLB01, we found key genes of the type VI secretion system (T6SS) [19], which is considered a virulence factor in many *Proteobacteria* [28]. Moreover, these genes for floc formation, including an operon for the biosynthesis of exopolysaccharides and an operon containing genes of PEP-CTERM/XrtA system glycosyltransferase, the PEP-CTERM system histidine kinase PrsK, and PEP-CTERM-box response regulator transcription factor prsR are homologous to the described genes for floc formation in *Zoogloea resiniphila* [29].

It was shown that the genome of the strain *Janthinobacterium* sp. SLB01 contains virulence factors [21]. Therefore, we conducted a test of the effect of this strain on the cell culture of primmorphs from the healthy sponge *L. baikalensis* including reisolation, sequencing, and analysis of the genome of *Janthinobacterium* sp. The pathogenicity of the strain *Janthinobacterium* sp. SLB01 for Baikal sponges and primmorphs is not known. The purpose of this study is to show that the strain *Janthinobacterium* sp. SLB01 isolated from the diseased sponge *L. baikalensis* is a pathogen for the cell culture of primmorphs. This research will help clarify the etiological factors of the diseases and mortality among Baikal sponges.

## 2. Materials and Methods

### 2.1. Sampling of Sponges and Cell Culture of Primmorphs

Specimens of the healthy sponge *L. baikalensis* Pallas, 1776 (Demospongiae, Haplosclerida, Spongillida, Lubomirskiidae) were collected by scuba divers in individual containers from Lake Baikal in the Olkhon Gate Strait, Central Siberia, Russia (53°02′21″ N; 106°57′37″ E), at a depth of 10 m (water temperature 3–4 °C). The cell cultures of primmorphs were obtained via the mechanical dissociation of cells according to the previously described technique [30]. A clean sponge sample was crushed, and the obtained cell suspension was subsequently filtered through sterile 200-, 100-, and 29 μm nylon meshes. The gel-like suspension was diluted 10-fold with Baikal water, placed in a refrigerator, and stored for 3 min at 3–6 °C until a dense precipitate formed. Healthy primmorphs were placed into 200–500 mL cultural bottles (Nalge Nunc International, Rochester, NY, USA). Cell cultures of primmorphs were cultivated in natural Baikal water (NBW) at 3–4 °C under illumination with a light intensity of 47 lx or 0.069 W with a 12 h cycle of day and night changes for a month. The cell culture of primmorphs was then used for experimental infection. 

### 2.2. Bacteria Isolation

In this study, we used the strain *Janthinobacterium* sp. SLB01 isolated from a sample of the diseased sponge *L. baikalensis*, collected in Lake Baikal, Central Siberia, Russia [19] for subsequent experimental infection. Healthy primmorphs (diameters of 2–4 mm) were transferred to 24-well plates (Nalge Nunc International, Rochester, NY, USA), with one piece per well in 2 mL of NBW, and infected with the *Janthinobacterium* sp. strain SLB01 with an initial dose of bacteria of 2.5 × 10^4^ CFU/mL in 50 μL. The infection was repeated at least three times. The infected primmorphs were cultivated at 3–6 °C with a 12-h day and night cycle for 14 days. During the infection of primmorphs, observations were carried out with daily descriptions and sampling for DNA isolation and sequencing. The cell suspension from the infected primmorphs was then homogenized and filtered using an MF-Millipore membrane filter of 0.45 µm pore size (Merck, Zug, Switzerland), and 10 µL was transferred to the nutrient medium. The bacteria were cultured on a nutrient media with R2A (0.05% yeast extract, 0.05% tryptone, 0.05% casamino acids, 0.05% dextrose, 0.05% soluble starch, 0.03% sodium pyruvate, 1.7 mM K_2_HPO_4_, 0.2 mM MgSO_4_, final pH 7.2 adjusted with crystalline K_2_HPO_4_ or KH_2_PO_4_) agar plates (Merck KGaA, Darmstadt, Germany) at pH 7.2. The dishes were inoculated with three repetitions and cultivated at a temperature of 22 °C for 5 days, with the growth of the strain observed daily.

### 2.3. Microscopy

We observed daily changes in infected cell cultures of primmorphs with *Janthinobacterium* sp. strain SLB01 over 14 days. The samples were stained with a NucBlue Live ReadyProbes reagent (Thermo Fisher Scientific Inc., Waltham, MA, USA). The cell morphology was determined via light microscopy on an Axio Imager Z2 microscope (Zeiss, Oberkochen, Germany) equipped with fluorescence optics (self-regulating, blue HBO 100 filter, 358/493 nm excitation, 463/520 nm emission). 

The samples were prepared for Scanning Electron Microscopy (SEM) analysis. Fixation was performed according to the following procedure: pre-fixation in 1% OsO₄ (10 min), washing in a cacodylate buffer (30 mM, pH 7.9), (10 min), fixation in 1.5% glutaraldehyde solution on a cacodylate buffer (30 mM, pH 7.9), (1 h), washing in a cacodylate buffer (30 mM, pH 7.9), (30 min), postfixation in 1% OsO₄ solution on a cacodylate buffer (30 mM, pH 7.9) (2 h), and washing in filtered Baikal water for 15 min at room temperature followed by dehydration in a graded ethanol series. The specimens were placed into SEM stubs, dried to a critical point, and coated with liquid carbon dioxide (BalTec CPD 030) using a Cressington 308 UHR sputter coater before examination under a Sigma series scanning electron microscope (Zeiss, Oberkochen, Germany) operating at 5.0 190 kV.

The samples were prepared for Transmission Electron Microscope (TEM) analysis. We took both healthy primmorphs and infected primmorphs for 24 h, 3 and 7 days with the strain *Jantinobacterium* sp. SLB01. The samples were fixed with 2.5% glutaraldehyde in a 0.1 M cacodyllate buffer (pH 7.2) for 24 h at 4 °C. The material was then washed in a 0.1 M cacodyllate buffer (pH 7.2) 3 times for 1 h, and 1% OsO_4_ diluted in 0.1 M cacodyllate buffer (pH 7.2) was fixed for 30 min. After washing from the fixative in distilled water (3 times for 30 min), the material was dehydrated in a series of increasing concentrations of ethanol and acetone. Next, the material was embedded in a mixture of Epon and Araldite (Sigma, Missouri, MO, USA). Semi-thin and ultra-thin sections were made using a Leica UC7 ultramicrotome (Leica Microsystems, Wetzlar, Germany). Ultrathin sections were contrasted with a 0.5% aqueous solution of uranyl acetate (20 min) and Reynolds lead citrate (10 min). Ultrathin sections were analyzed using a Libra 200 FE transmission electron microscope (Carl Zeiss, Oberkochen, Germany) and a Libra 120 (Carl Zeiss, Oberkochen, Germany).

### 2.4. Genome Sequencing

Genomic DNA for full-genome sequencing was isolated following a standard bacterial DNA isolation cetyltrimethylammonium bromide (CTAB) protocol (https://jgi.doe.gov/wp-content/uploads/2014/02/JGI-Bacterial-DNA-isolation-CTAB-Protocol-2012.pdf, accessed on 10 September 2020). The sequence library was prepared with a Nextera XT DNA library preparation kit (Illumina, San Diego, CA, USA). Genomes were sequenced on the Illumina MiSeq platform using v2 paired-end chemistry (2 × 250 bp, 12,099,942 reads total).

### 2.5. Genome Assembly, Annotation and Phylogenetic Relationship

Raw read error correction and filtering with FastP tool were performed with default settings [31]. Genomes were assembled with SPAdes version 3.11.0 [32] using the default settings. Contigs from draft assembly with a length of more than 10 Kbp were scaffolded with Ragout version 2.2 with default settings [33] (https://github.com/fenderglass/Ragout, accessed on 9 March 2021) using *Janthinobacterium* sp. LM6 chromosome (GenBank accession no. CP019510) as the reference. We used the same software set for genome assembly and annotation to prevent genome variations depending on reference and assembly software versions. Although the prokaryotic genome annotation pipeline (PGAP) version for annotating the genome *Janthinobacterium* sp. strain SLB01 was 4.13 (used when the genome was released in NCBI RefSeq), to annotate *Janthinobacterium* sp. PLB02, we used the newest available NCBI PGAP version (5.1), which can annotate more genes because its database is richer. Gene annotations were performed using PGAP (https://github.com/ncbi/pgap, accessed on 9 March 2021). Core genome construction was accomplished with Roary version 3.13.0 using default settings [34]. Genome completeness analysis was performed with benchmarking universal single-copy orthologs (BUSCO) version 5.0.0 using the dataset “burkholderiales_odb10” [35]. Strains of *Janthinobacterium* spp. identification was carried out via phylogenetic analysis with PhyloPhlAn 3.0 [36] based on a comparison of 400 universal marker genes (a maximum-likelihood method) [37] using the “supermatrix_aa” and “low diversity” modes with the “phylophlan” database. We acquired 10 closely related strains of *Janthinobacterium* by 16S rRNA from the Basic Local Alignment Search Tool (BLAST/-NCBI) to build a phylogenetic tree.

### 2.6. Statistical Analysis

All of the infection experiments were performed at least three times. The data were reported as the means ± standard deviation (SD). A statistical analysis was then carried out (single-factor (ANOVA) followed by Tukey’s multiple range test) using the SPSS.16 software. Differences in mean values were considered significant at *p* < 0.05.

## 3. Results

### 3.1. Bacterial Isolation and Microscopy

**Figure 1:**
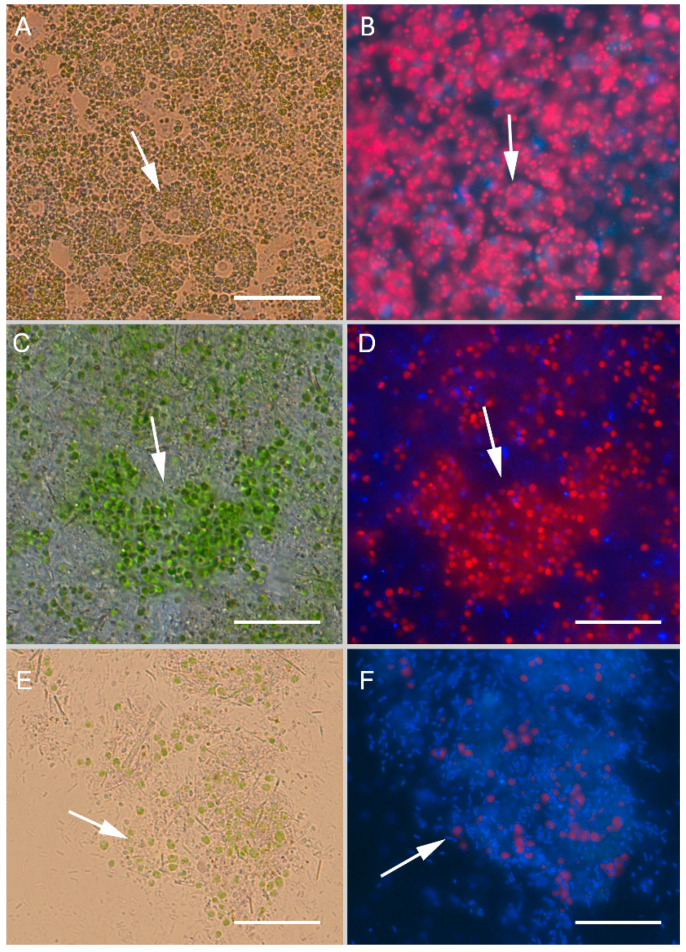
Light and fluorescence images of the cell culture of primmorphs of sponge *L. baikalensis*: (**A**,**B**) Green microalgae located within arrow amoebocytes in the healthy cell cultures of primmorphs. The autofluorescence of chlorophyll-containing intracellular microalgae is shown in red (**C**,**D**) chaotic arrangements of green microalgae and increases in bacteria in primmorphs infected with *Janthinobacterium* sp. strain SLB01 on day 3 of cultivation. Bacteria are shown with a blue color (indicated by the arrows); (**E**,**F**) the primmorphs infected with *Janthinobacterium* sp. strain SLB01 on day 7 of bacteria cultivation (blue color; shown by arrows). The samples of primmorphs were stained with the NucBlue Live ReadyProbes reagent for fluorescence microscopy. Scale bars: 10 µm.

We used a model cell culture of healthy primmorphs of sponge *L. baikalensis* for experimental infection with the *Janthinobacterium* sp. strain SLB01 isolated from a diseased sponge, *L. baikalensis,* to determine the bacterial pathogenicity. The healthy primmorphs were bright green in color and with bright red autofluorescence of chlorophyll in the cells due to the presence of green symbiotic microalgae belonging to the taxon Chlorophyta in their composition. We observed that the cells of the sponges contained a strict arrangement of symbiotic microalgae in the amoebocytes of the uninfected primmorphs (Figure 1A,B). 

A completely different picture was observed in primmorphs infected with the *Janthinobacterium* sp. strain SLB01. Dirty scurf, a fetid odor, and biofilm formation were observed in the infected cultures, which were likely associated with the growth of bacteria. The primmorphs lost their green color after infection with the strain *Janthinobacterium* sp. SLB01 (Figure 1C). We observed the destroyed cells of amoebocytes and a chaotic arrangement and adhesion of microalgae. On the third day of cultivation of primmorphs infected with *Janthinobacterium* sp. strain SLB01, we observed the suppression of autofluorescence (Figure 1D). After 7 days of cultivation of the infected culture of primmorphs, we observed dead cells of sponges and a chaotic arrangement of microalgae with an increase in the number of short rod-shaped bacteria (Figure 1E,F). The loss of chlorophyll autofluorescence and the death of microalgae were observed in all experimental samples. 

**Figure 2:**
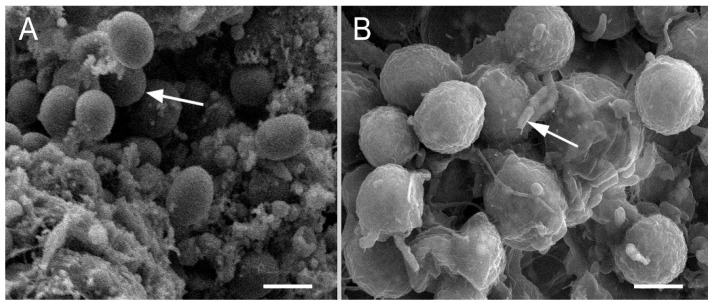
SEM images of cell cultures of primmorphs: (**A**) the healthy primmorphs not infected with *Janthinobacterium* sp. strain SLB01 (shown by arrows); (**B**) the primmorphs infected with *Janthinobacterium* sp. strain SLB01. The formation of microbial biofilm with short rod-shaped bacteria was observed on the 14th day of cultivation on the surfaces of symbiotic microalgae. Scale bars: 2 µm.

Using SEM, we found that the microalgae contained spheroidal cells 2.5–3.0 µm in diameter with a clean cell wall in the healthy primmorphs (Figure 2A).

However, we experimentally observed the interaction of bacteria with host cells in the primmorphs infected with *Janthinobacterium* sp. strain SLB01 (Figure 2B). The squamous epithelium was destroyed, and the symbiotic microalgae were packed entirely in a thick microbial biofilm with short rod-shaped bacteria (Figure 2B). 

**Figure 3:**
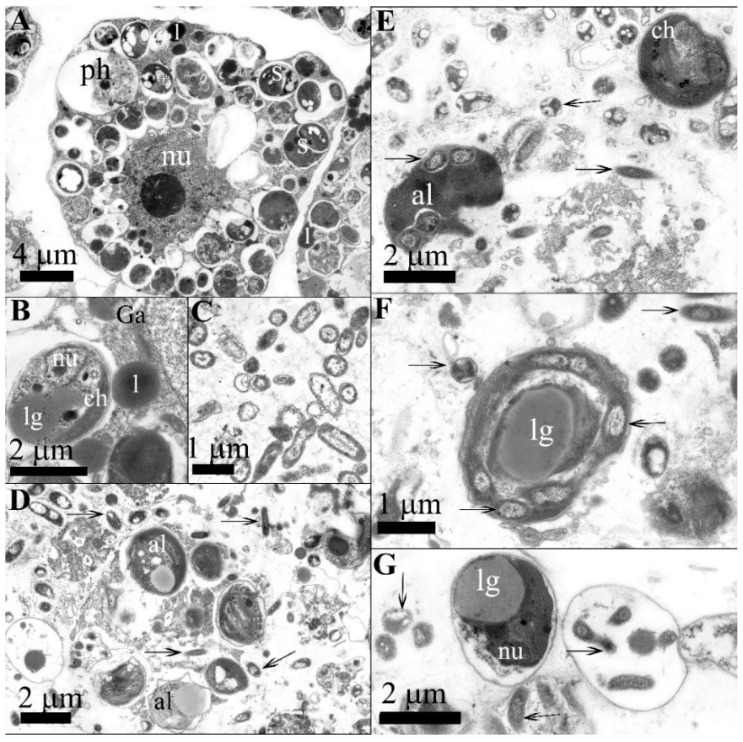
Ultrastructural analysis of the cell culture of primmorphs in the sponge *L. baikalensis* and *Janthinobacterium* sp. strain SLB01: (**A**) the amebocyte of the healthy primmorph with symbiotic microalgae; (**B**) symbiosome in the amebocyte of the healthy primmorphs with the microalgae containing a nucleus, chloroplast, and inclusions, including osmiophilic lipid granules and starch grains (marked with asterisks); (**C**) the *Janthinobacterium* sp. strain SLB01 bacterial culture; (**D**) the primmorphs of the sponge *L. baikalensis* one day after infection and the presence of Gram-negative bacteria (marked with black arrows) in the mesohyl and amebocytes; (**E**,**F**) bacteria form colonies in microalgae cells and cells undergoing lysis and bacteria connected by a network of contacts on the 7th day of infection; (**G**) the cells of the microalgae undergoing lysis and bacteria penetrating free-lying microalgae and forming colonies. Legend: al–microalgae cell; ch–chloroplast; l–primary lysosome, lg–lipid granule; nu–nucleus; ph–phagosome.

We observed an interaction of bacteria with the host cells in the primmorphs and symbiotic microalgae infected with the *Janthinobacterium* sp. strain SLB01 through the use of ultrastructural analysis (Figure 3).

We found that amoebocyte cells were filled with green symbiotic microalgae in the healthy primmorphs (Figure 3A). The amoebocyte cells were up to 20 µm in diameter, containing a nucleus with a prominent nucleolus. The cytoplasm of amoebocytes contains dictyosomes of the Golgi apparatus and cisterns of the endoplasmic reticulum. A distinctive feature of amoebocytes is the presence in the cytoplasm of specialized vacuoles—symbiosomes with symbiotic microalgae representatives of the *Chlorophyceae* family enclosed in them. Microalgal cells (2.5–3.0 µm in diameter) have a thin electron-dense polysaccharide envelope separated from the cell’s outer membrane by a narrow supramembrane space (Figure 3B). There is also a chloroplast, which can contain electron-transparent inclusions that are, most notably, starch grains. Granules are often present between the thylakoid membranes and directly in the cytoplasm of the microalgae (Figure 3B). In addition, it was noted that no bacteria were found in the mesohyl of healthy primmorphs. In the mesohyl of infected cell cultures, rod-shaped bacteria were found in the primmorphs 24 h after infection (Figure 3D). The structure of the bacteria had an enlarged folded outer membrane and there was an electron-transparent halo observed around the bacterial cells which indicated their ability to lyse the surrounding components of the extracellular matrix. In addition to mesohyl, bacteria were present in amoebocytes, which penetrated via phagocytosis. The system of intracellular membranes was destroyed, and vacuolization was enhanced. The symbiosomes with microalgae enclosed in them were preserved in the cytoplasm of amoebocytes. Some symbiotic microalgae left the host cells and were located within the extracellular matrix. The destruction processes of cells of primmorphs reached the terminal stage on day 7 after infection (Figure 3E). The microalgae were located directly in the mesoglea, where they became available for the action of bacteria on day 7 after the start of the infection. The cytoplasm in the cells of primmorphs was fragmented, resulting in the absence of whole functionally active cells. We observed that the extracellular matrix also contained symbiotic microalgae infected with bacteria, which, due to division, subsequently formed colonies of bacteria united by contact processes (Figure 3F). The formation of bacterial colonies was accompanied by the lysis of the components of the microalgal cytoplasm, with the presence of a polysaccharide shell enclosing bacteria (Figure 3G). Thus, as the infection progressed, the cells of primmorphs became lysed, and microalgae were localized mainly in the mesohyl. During this period, the cells became infected with bacteria; this eventually led to the formation of bacterial colonies inside the microalgal shell on day 7 post-infection.

The strain *Janthinobacterium* sp. PLB02 was isolated from a cell culture of primmorphs infected with the *Janthinobacterium* sp. strain SLB01. The bacteria were rod-shaped, motile, and aerobic; in addition, the purple pigment violacein appeared on the second day. A morphological analysis showed that the bacteria have short rods up to 2.0 µm long and 0.3 µm in diameter, with a two-layer outer membrane typical of Gram-negative bacteria (Figure 3C). The cytoplasm, in most cases, was granular, flagellated and electron-dense, with a well-defined nucleoid zone.

### 3.2. Comparison of Genomes of Janthinobacterium spp. Strains

In the present study, we compared the genomic contents of two strains: *Janthinobacterium* sp. SLB01 and reisolated *Janthinobacterium* sp. PLB02. We assembled the genome of the *Janthinobacterium* sp. strain PLB02 from sequence data the same way as was done for the *Janthinobacterium* sp. SLB01 strain. The final genome assembly statistics of the raw read count, genome size, number of genes, pseudogenes, protein-coding sequences, tRNA noncoding RNA, and references to genome reports are presented in Table 1.

A genome completeness analysis with benchmarking universal single-copy orthologs (BUSCO) [35] showed results for the strains *Janthinobacterium* spp. SLB01 and PLB02, with 99.1% complete (not fragmented) and 0.9% missing BUSCOs. The fully assembled genomes included 6,467,981 bp for strain *Janthinobacterium* sp. SLB01 and 6,417,505 bp for strain *Janthinobacterium* sp. PLB02, and exhibited similar G+C contents (62.63% and 62.65%, respectively). Genome annotation with PGAP revealed 5643 genes (5502 protein-coding) for strain SLB01 and 5651 (5510 protein-coding) for strain PLB02, as shown in Table 1. We compared the genomic contents of the *Janthinobacterium* sp. strain SLB01 and *Janthinobacterium* sp. strain PLB02 with the data of Roary [34] and found that most of the genes were the same (with a homology of more than 99%). 

### 3.3. Phylogenetic Relationship

**Figure 4:**
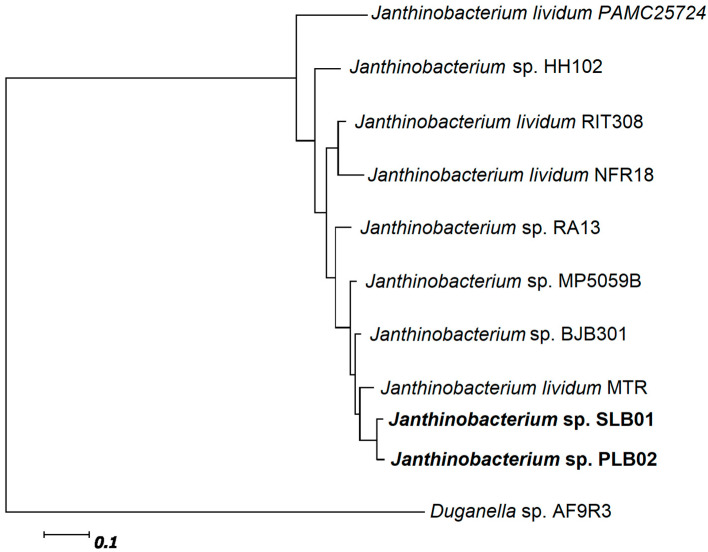
Phylogenetic tree of strains *Janthinobacterium* sp. SLB01 and *Janthinobacterium* sp. PLB02, highlighted in bold, with closely related species. The tree was built based on 400 universal marker genes.

We built a phylogenetic tree for the *Janthinobacterium* species to compare the genomic features of both strains—*Janthinobacterium* spp. SLB01 and PLB02—with closer species [38]. Strain *Janthinobacterium* sp. PLB02 showed the highest phylogenetic affiliation to strain *Janthinobacterium* sp. SLB01 of phylum *Proteobacterium* from the family *Oxalobacteraceae* (Figure 4). 

The result of the phylogenetic tree based on 400 universal marker genes using PhyloPhlAn (a maximum-likelihood method) [35] showed that the genomes of *Janthinobacterium* sp. SLB01 and *Janthinobacterium* sp. PLB02 are homologous to each other and very closely related to the psychrotolerant strain *J. lividum* MTR (Figure 4).

### 3.4. Analysis of the Virulence Genes

**Figure 5:**
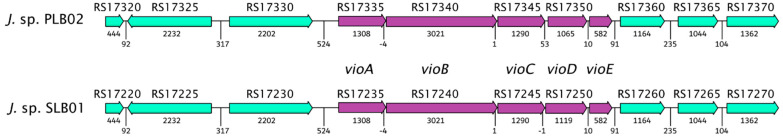
Schematic diagram of the genetic organization of vioABCDE loci and flanking regions in the *Janthinobacterium* sp. strain PLB02 (locus prefix J3P46) and *Janthinobacterium* sp. strain SLB01 (locus prefix F3B38). Loci J3P46_RS17320 (F3B38_RS17220) encoded DUF411 domain-containing protein in the strains *Janthinobacterium* spp. PLB02 and SLB01, respectively; J3P46_RS17325 (F3B38_RS17225)–NADP-dependent isocitrate dehydrogenase; J3P46_RS17330 (F3B38_RS17230)–TonB-dependent siderophore receptor; J3P46_RS17335 (F3B38_RS17235)–FAD-dependent oxidoreductase (VioA); J3P46_RS17340 (F3B38_RS17240)–iminophenyl-pyruvate dimer synthase VioB; J3P46_RS17345 (F3B38_RS17245)–FAD-dependent monooxygenase (VioC); J3P46_RS17350 (F3B38_RS17250)–tryptophan hydroxylase (VioD); J3P46_RS17355 (F3B38_RS17255)–violacein biosynthesis enzyme VioE; J3P46_RS17360 (F3B38_RS17260)–MFS transporter; J3P46_RS17365 (F3B38_RS17265)–helix-turn-helix domain-containing protein; J3P46_RS17370 (F3B38_RS17270)–Vps62-related protein. Genes that are displayed with arrows and Loci tags are indicated with a regular font, and gene names are indicated with an italic font. Violacein operons are displayed with violet color full and flanking regions are displayed with green color.

We compared the obtained genomes of strains of the *Janthinobacterium* sp. PLB02 and *Janthinobacterium* sp. SLB01 with each other and analyzed the coding virulence proteins and key genes such as the genes of violacein, floc formation, and the type VI secretion system [21]. We found that the strains were 100% homologous to each other in terms of virulence factors. Earlier, we showed that the strain *Janthinobacterium* sp. SLB01 was able to produce violacein and contained the violacein synthesis operon vioABCDE [21,22]. Isolated from primmorphs, the strain *Janthinobacterium* sp. PLB02 also produced the pigment violacein, and the genome contained the violacein synthesis operon vioABCDE. The flanking regions for gene coordinates and locus names are presented in Figure 5 and Table 2.

We found 100% homology of the strains *Janthinobacterium* spp. SLB01 and PLB02. We used the generally accepted names of the genes of the violacein synthesis operon vioABCDE instead of the annotated names when comparing their genomes (Table 2).

**Figure 6:**
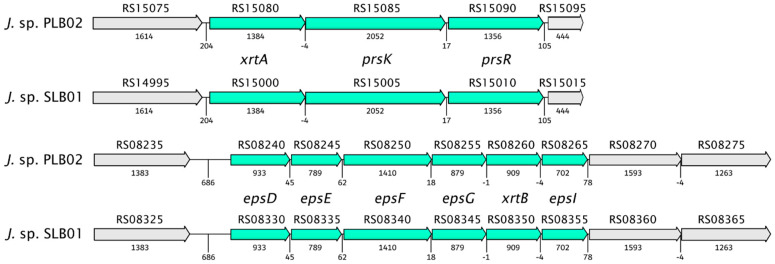
Schematic diagram of the genetic organization of *Janthinobacterium* sp. strain PLB02 and *Janthinobacterium* sp. strain SLB01 gene clusters required for floc formation and flanking regions for PEP-CTERM, exosortase, and exopolysaccharides (EPS) synthesis. Arrows indicate genes, and the directions of the arrows represent the direction of transcription of the genes in the genome. Gene names are written with an italic font. Loci J3P46_RS08260, (F3B38_RS08350)–exosortase B; J3P46_RS15075 (F3B38_RS14995)–L-aspartate oxidase; J3P46_RS15080, J3P46_RS15085 and J3P46_RS15090 (F3B38_RS15000, F3B38_RS15005 and F3B38_RS15010)–PEP-CTERM/XrtA system glycosyltransferase (XrtA), histidine kinase (PrsK) and response regulator transcription factor (PrsR); J3P46_RS08240–J3P46_RS08255 and J3P46_RS08265 (F3B38_RS08330–F3B38_RS08345 and F3B38_RS08355) encoding proteins EpsD, EpsE, EpsF, EpsG and EpsI in the strains *J.* sp. PLB02 and *J.* sp. SLB01, respectively. Gene clusters required for floc formation are displayed with the semitransparent green color and flanking regions are displayed with the gray color.

Our previous study discovered gene clusters involved in floc formation [21,22]. We found that the clusters of genes of the *Janthinobacterium* sp. strain PLB02 have 100% structural similarity to the genome of the strain *Janthinobacterium* sp. SLB01 (Figure 6). 

Previously, we showed that the *Janthinobacterium* sp. strain SLB01 formed a strong biofilm rich in exopolysaccharides (EPS) in the stationary phase [21,22]. Interestingly, the isolated strain *Janthinobacterium* sp. PLB02 also formed a strong biofilm. The strain produced floc and biofilm via exopolysaccharide biosynthesis and PEP-CTERM/XrtA protein expression. A genome analysis showed that the *Janthinobacterium* sp. strain PLB02 has all the necessary gene cassettes for flocculation, similar to strain *Janthinobacterium* sp. SLB01. Both genomes contain the system glycosyltransferase putative exosortase XrtA (previously called EpsH), the PEP-CTERM system histidine kinase PrsK, the PEP-CTERM system associated sugar transferase, the sensor histidine kinase of a two-component system, and the PEP-CTERM-box response regulator transcription factor PrsR. Localization, annotation, and identity percentages of these genes are presented in Table 3. 

Moreover, we analyzed the type VI secretion system (T6SS) as the primary virulence factor in the genome of the strain *Janthinobacterium* sp. PLB02 and found that the genes of both strains were 100% identical to each other. As in the strain Janthinobacterium sp. SLB01, the genome of the isolated strain *Janthinobacterium* sp. PLB02 contained all three categories of genes required for the type VI secretion system to function. The isolated strain *Janthinobacterium* sp. PLB02 was identical to strain *Janthinobacterium* sp. SLB01.

## 4. Discussion

In this study, we showed that the strain *Janthinobacterium* sp. SLB01 isolated from a diseased sponge *L. baikalensis* and infected cell culture of primmorphs is the same and that the genomes of the strains are identical. The strains *Janthinobacterium* sp. SLB01 and *Janthinobacterium* sp. PLB02 are pathogens for cell cultures of primmorphs and the sponge *L. baikalensis*. After experimental infection of the cell culture of primmorphs, we found that short rod-shaped bacteria of the strain *Janthinobacterium* sp. SLB01 grew quickly and parasitized sponge cells and their symbiotic microalgae. We detected the death of the symbiotic microalgae (Chlorophyta) and the sponge cells in the infected primmorphs, as well as increased bacteria counts.

The bacteria *Janthinobacterium* sp. was found in the mesohyl of cell cultures of primmorphs 24 h after infection and was able to lyse the primmorph cells. The characteristic features of the structure of *Janthinobacterium* sp. during the development of the infectious process included the presence of a folding outer membrane, an increase in the periplasm, and an electron-transparent zone of lysis around the bacterial cells. It is known that the outer cell membrane and periplasm of Gram-negative bacteria serve as the compartments responsible for the production of secondary metabolites, including proteolytic enzymes and other factors of bacterial cell virulence [39,40]. An increase in the surface area of the outer membrane and the volume of the periplasm in *Janthinobacterium* sp. over the course of infection indicates the activation of processes aimed at realizing their pathogenic potential. We observed that the *Janthinobacterium* sp. penetrated the cytoplasm of microalgae and lysed their contents, using nutrients for growth, division, and the formation of colonies of the bacteria. The infection process progressed in the sponge cells of the primmorphs and the microalgae and reached the terminal stage on the day 7 of infection, thus indicating a rapid course of the pathogenic process. We observed the destruction of the photosynthetic apparatus, the loss of chlorophyll autofluorescence, and the death of symbiotic microalgae in all the infected primmorphs. 

Earlier, we showed that the cell culture of the primmorphs of healthy sponge *L. baikalensis* is identical to that of sponges, and can be used as a model system for studying the diseases of Baikal sponges [18]. Here, we showed that during the experimental infection of the cell culture of primmorphs with the strain *Janthinobacterium* sp. SLB01, the bacteria attacked eukaryotic cells of the microalgae and then acquired the released nutrients after cell lysis (Figure 3F,G).

A comparison of the two genomes from *Janthinobacterium* sp. SLB01 and *Janthinobacterium* sp. PLB02 isolated from the diseased sponge and infected cell cultures of the primmorphs showed that genomes of these bacteria have identical genomic content. The genome sizes, gene counts, and G+C content were very close. The genome size of *Janthinobacterium* strains slightly differed due to the number of Ns (unknown nucleotides) after the scaffolding procedure. Moreover, we found that these species are rod-shaped Gram-negative bacteria that produce violacein, a compound with antimicrobial and antiviral properties that is toxic to eukaryotic cells [41]. The isolated bacteria *Janthinobacterium* sp. PLB02 can colonize the space and possibly suppress the grown microalgae with the pigment violacein. This pigment production was observed in the infected primmorphs, and all the genes (operon vioABCDE) were present in its genome. We identified five genes encoding VioA, VioB, VioC, VioD, and VioE proteins related to violacein biosynthesis similar to those identified in published *Janthinobacterium* sp. SLB01. Earlier, we observed that one essential strategy of the *Jantinobacterium* sp. strain SLB01 is the secretion of virulence factors through the cell membranes of the victim to achieve a potential target [21,22]. In addition, an identical T6SS secretion system of the strain *Jantinobacterium* sp. SLB01 was found in the isolated *Janthinobacterium* sp. PLB02. Both strains’ genomes contained all three categories of genes required for the function of type T6SS [42,43]. 

Bacterial strains *Janthinobacterium* spp. SLB01 and PLB02, based on a comparison of complete genomes, showed similarity with the strain *J. lividum* MTR. Interestingly, *J. lividum* either caused necrosis on mushroom tissue blocks or colonized the skin of some amphibians, conferring protection against fungal pathogens [27,44]. In addition, isolated bacteria also produced floc formation and strong biofilm in the stationary phase. When cultivating the strains *Janthinobacterium* sp. SLB01 and *Janthinobacterium* sp. PLB02, we observed biofilm and floc formation in the diseased sponges and the infected cell cultures of primmorphs of *L. baikalensis*. A genomic analysis of the two strains found RpoN, PepA, XrtA, PrsK, and PrsR gene clusters present in the formation of floc and 100% similarity between the strains (Table 2). 

Using an ultrastructural analysis, we found that the symbiotic microalgae were completely enclosed in a thick microbial biofilm during the infection of primmorphs with the strain *Jantinobacterium* sp. SLB01 (Figure 2B). Moreover, on day 7 after infection, it was discovered that the formation of bacterial colonies was accompanied by utilization of the components of the microalgal cytoplasm; there remained only a polysaccharide shell with bacteria enclosed in it (Figure 3G). Thus, floc formation and biofilm can negatively affect the physiology of the life of the host (sponge *L. baikalensis*) due to clogging of the pores. These negative effects of biofouling on the functioning of the filter-feeding marine sponge *Halisarca caerulea* were previously reported [45]. Exopolysaccharides (EPS) are known to be the main component of the biofilm produced by the species of *Oxalobacteraceae* [46]. 

It is known that the family *Oxalobacteraceae* is characterized by the presence of extremely ecologically diverse species of microorganisms and contains environmental saprophytic organisms, phytopathogens, and opportunistic pathogens, including those common in freshwater ecosystems [47]. The genomes of many environmental isolates of *Janthinobacterium* from ice, water, sediments, and soils were sequenced [25,26,27], but strains of *Janthinobacterium* sp. strain SLB01 and the new *Janthinobacterium* sp. strain PLB02 from the Baikal sponge and cell culture of primmorphs were isolated in this study for the first time.

The disease and mass mortality of sponges and corals have been observed worldwide in the marine environment in recent years [48,49,50,51,52], and corresponding die-off events threaten overall sponge-associated biodiversity [53,54,55,56]. These changes in sponge–microbe interactions appear to be associated with climate change and the occurrence of opportunistic infections resulting from changes in water temperature caused by global warming, light intensity, and salinity [57,58,59,60,61,62]. Previously, Webster et al., presented a description of the pathogenic bacterial strain NW4327 isolated from an infected marine sponge *Rhopaloeides odorabile* in the Great Barrier Reef [63]. Choudhury et al. reported the isolation of the pathogenic bacterial strain of *Pseudoalteromonas agarivorans* found in diseased sea sponges with pathogenicity genes [64].

Thus, in this study, we sought to reproduce Koch’s postulates with a cell culture of primmorphs. The present study is the first of its kind. We were able to isolate the new strain of *Janthinobacterium* sp. PLB02 after infecting a cell culture of primmorphs using the strain *Janthinobacterium* sp. SLB01 isolated from a diseased sponge *L. baikalensis*. We found that the strains are the same and have virulence factors in their genomes. We showed interactions of the *Janthinobacterium* sp., marking this species as a potential pathogen for cell cultures of primmorphs of the Baikal sponge *L. baikalensis*. The results of this study will help expand our understanding of microbial interactions in the development of disease and the death of Baikal sponges.

## Figures and Tables

**Table 1:** General genome features of strains *Janthinobacterium* spp. SLB01 and PLB02.

Property	*Janthinobacterium* sp. Strain PLB02	*Janthinobacterium* sp. Strain SLB01
Classification	*Proteobacteria*, *Betaproteobacteria*, *Burkholderiales*, *Oxalobacteraceae*, *Janthinobacterium*	
Gram stain	negative	
Cell shape	rod shape	
Motility	twitching	
Temperature	15–22 °C	
Pigmentation	purple	
Host	primmorphs *L. baikalensis* sponge *L. baikalensis*	
Sequencing method	Illumina MiSeq (2 × 250)	
Raw reads	7,471,704	12,099,942 *
Genome coverage	180x	180x
GenBank accession number	NZ_CP071520.1	VZAB00000000.1
Genome size, bp	6,417,505	6,467,981
Assembly method	SPAdes version 3.11.0	
Annotation method	NCBI Prokaryotic Genome Annotation Pipeline	
Number of contigs	1	2
GC content	62.65%	62.63%
Number of genes	5651	5643
Protein-coding sequences	5510	5502
tRNAs	76	76
Noncoding RNAs	4	4
Pseudogenes	57	56

* Before quality filtering. The sequence library was generated from DNA using an Illumina Nextera XT DNA sample preparation kit. Whole-genome sequencing was performed using the Illumina MiSeq platform with paired-end chemistry (2 × 250 bp).

**Table 2:** Violacein production genes localization in genomes of the strains *Janthinobacterium* sp. SLB01 and *Janthinobacterium* sp. PLB02.

Gene Name	Locus Tag		Begin		End		Homology %	Length, bp	
Strain	SLB01	PLB02	SLB01	PLB02	SLB01	PLB02		SLB01	PLB02
vioA	F3B38_RS17235	J3P46_17335	1353472	3909260	1354779	3910567	100	1308	1308
vioB	F3B38_RS17240	J3P46_17340	1354776	3910564	1357796	3913584	100	3021	3021
vioC	F3B38_RS17245	J3P46_17345	1357798	3913586	1359087	3914875	100	1290	1290
vioD	F3B38_RS17250	J3P46_17350	1359087	3914929	1360205	3915993	100	1119	1065
vioE	F3B38_RS17255	J3P46_17355	1360216	3916004	1360216	3916585	100	582	582

**Table 3:** Floc formation loci in the genomes of the strains *Janthinobacterium* spp. SLB01 and PLB02.

Component	Locus Tag (PLB02)	Length, aa (PLB02)	Locus Tag (SLB01)	Length, aa (SLB01)	Identity %	Annotation
RpoN	J3P46_RS03515	1479	F3B38_RS03495	1479	100	RNA polymerase factor sigma-54 (RpoN)
PepA	J3P46_RS08815	1494	F3B38_RS08900	1494	100	leucyl aminopeptidase (PepA Zooglea sp.) Over expression could circumvent the requirement of rpoN, prsK and prsR for the floc-forming phenotype by fixing the exopolysaccharides to bacterial cells.
XrtA	J3P46_RS15080	1389	F3B38_RS15000	1389	100	TIGR03013 family PEP-CTERM/XrtA system glycosyltransferase putative exosortase XrtA (previously called EpsH)
PrsK	J3P46_RS15085	2052	F3B38_RS15005	2052	100	PEP-CTERM system histidine kinase PrsK Sensor histidine kinase of a two-component system
PrsR	J3P46_RS15090	1356	F3B38_RS15010	1356	100	PEP-CTERM-box response regulator transcription factor PrsR Sensor histidine kinase of a two-component system

## Data Availability

This full-genome shotgun project was deposited in GenBank and published with accession number NZ_CP071520.1.
